# The trends of aquacultural nitrogen budget and its environmental implications in China

**DOI:** 10.1038/s41598-018-29214-y

**Published:** 2018-07-18

**Authors:** Zhibo Luo, Shanying Hu, Dingjiang Chen

**Affiliations:** Center for Industrial Ecology, Department of Chemical Engineering, Tsinghua University, Beijing, 100084 China

## Abstract

The rapid development of aquaculture has sustained aquatic food production but has also led to a host of environmental problems, ranging from eutrophication of aquatic ecosystems to global acidification. China has become the world’s largest producer and consumer of aquaculture products. Nitrogen is an essential nutrient in aquaculture ecosystems, and the quantitative environmental fate and impact of nitrogen during aquaculture processes have notable environmental consequences but have received little attention. Here, we established a nitrogen cycling model for China’s aquaculture ecosystem to investigate the creation and fate of reactive nitrogen over a decadal time scale. A nitrogen balance analysis showed that reactive nitrogen input in the aquaculture ecosystem increased from 9.43 Tg N yr^−1^ in 1978 to 18.54 Tg N yr^−1^ in 2015, while aquaculture production increased from 0.034 to 1.33 Tg N yr^−1^ during the same period. The environmental fate analysis showed that nitrogen emissions, accumulation, sediment deposition, and export into the oceans increased by 9.05-fold, 0.24-fold, 9.04-fold, and 2.56-fold, respectively. Finally, we investigated four scenarios representing different consumption levels of aquatic products and provided policy recommendations (larger aquaculture size, standardized aquaculture production model, nutritional element management and balanced dietary structure, etc.) on improved management practices in aquaculture ecosystems.

## Introduction

Rapid population and economic growth will greatly increase the demand for aquatic products^1^. As fishery yields from capture will be constrained by ecosystem productivity, the per capita consumption of aquatic products can only be maintained or increased if aquaculture makes an increasing contribution to the volume and stability of the supply of these products, particularly in a populous country such as China^2,3^. Aquaculture in China has made remarkable achievements in recent decades, as evidenced by China becoming the world’s largest producer, consumer, processor, and exporter of aquatic products^4^. This trend is largely due to China’s ever-expanding aquaculture sector, as most of its domestic fisheries are overexploited, but this development pattern has also caused serious ecological and environmental consequences^5,6^. Nitrogen (N) is an essential nutrient in aquaculture ecosystems, playing a central role not only in the food chain but also in environmental pollution (forms include NH_4_^+^, NH_3_, NO_2_^−^, HNO_2_, NO_3_^−^ and organic N). However, the fate and impact of reactive N (Nr) during the aquaculture process have not received sufficient attention on global and national scales other than the quantification of the Nr creations or Nr emissions during aquaculture. Recently initial attempts at aquaculture research in China mainly focus on various types of aquaculture systems (such as ponds, cages, reservoirs, inland rivers, lakes and coastal waters). For instance, Guo *et al*. demonstrated the assessment effects of cage culture on N and phosphorus dynamics in relation to fallowing in a shallow lake in China^7^. Xia *et al*. reported the N and phosphorus cycling in shrimp ponds in the Tailake region, China^8^. Gu *et al*. studied the aquaculture as one of the subsystems of the N cycle at the national level with several simple Nr flow (such as BNF, N deposition, fertilizer and crop) in aquaculture subsystem^9,10^. Zhang *et al*. performed a comprehensive review of the available case studies (51 peer-reviewed publications) on nutrient use efficiency in China’s aquaculture systems^11^. These estimates were based on local regions, simple N flow analysis or a limited number of case studies. An approach with a detailed, comprehensive perspective and top-down calculation for aquaculture N cycling in China at national scale is still lacking. The absence of this component from the study of human alteration of the N cycle is a serious concern, especially given the added pressure of population growth, the increasing demand for aquatic foods and environmental pollution problems. Accordingly, the study of the N cycle and its historical evolution in aquaculture ecosystems has important implications for deepening the N cycle at the national level, which will further influence the understanding of the cascade effect of N environmental impacts and N metabolism on food security.

**Figure 1:**
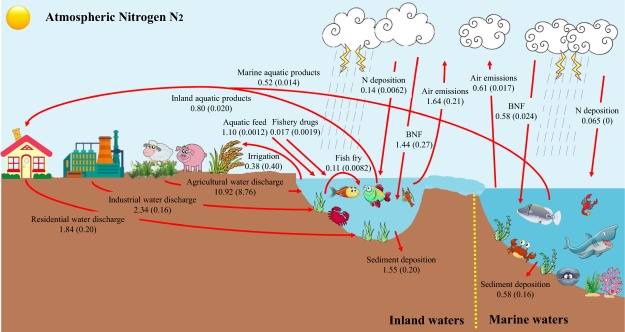
Schematic model of national Nr cycling in China’s aquaculture ecosystem. The unit is Tg N yr^−1^. The red lines represent Nr flow. The numbers in front of the parentheses represent the Nr flux in 2015; the numbers in parentheses represent the Nr flux in 1978. Only major flows are presented in this figure, but the flow description and data acquisition section in the methods section of this paper provide an overview of all simulated flows.

To address this issue, we establish an N cycling model for China’s aquaculture ecosystem (Fig. 1) and then calculate the total Nr input associated with aquatic food production on a yearly basis. A detailed Nr balance and environmental fate analysis in aquaculture ecosystems of China shows a serious issue arising from Nr being lost to the environment and negatively affecting ecosystems, such as Nr accumulation in water, which leads to eutrophication and loss of biodiversity, NH_3_ emissions and emissions of N_2_O. Then, we investigate future scenarios of aquaculture development driven by different levels of aquatic product consumption. Finally, we discuss the policy implications of mitigating the negative effects of Nr and improving aquaculture management efficiency while meeting the national demand for aquatic foods.

## Results

### An overview of the development of fisheries in China

**Figure 2:**
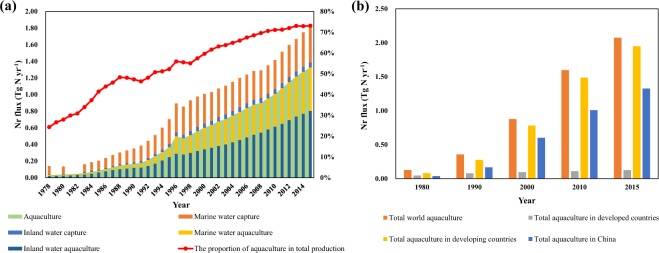
Changes in the production of Nr in China’s and the world’s fisheries. (**a**) Temporal variations in N production of aquaculture and capture in China from 1978 to 2015; (**b**) comparison of aquaculture N production among China, developed and developing countries.

The continued expansion of the world’s human population and economic development will increase future demand for fishery products. As an emerging economy and a highly populous country, China has seen rapid growth in the demand for aquatic products in recent years (Fig. 2). Total fishery production (including capture and aquaculture) in China grew from 0.14 Tg N yr^−1^ in 1978 to 1.81 Tg N yr^−1^ in 2015, an increase of nearly 12-fold (Fig. 2a). As marine fishery yield is constrained by natural ecosystem productivity, the increase in capture is relatively slow, with an average annual growth rate of 4.20%, while aquaculture has made an increasing contribution to meeting human consumption, increasing from 0.034 Tg N yr^−1^ to 1.33 Tg N yr^−1^, with an average annual growth rate of 10.42%. Due to the advantages of management efficiency in aquaculture, the share of production accounted for by aquaculture has increased from approximately one-quarter in 1978 to approximately three-quarters in 2015, ensuring a stable fish supply in China.

The rapid development of Chinese fisheries, especially aquaculture operations, is profoundly changing the industrial pattern of the world’s fisheries. In 1980, China’s aquaculture production (N component) accounted for 29.69% of world production, and by 2015, its share increased to 63.95% (Fig. 2b). The developed countries mainly rely on capture to meet their fish consumption, while the reverse is true for China. In 1990, China became the world’s largest aquaculture producer^12^. In subsequent decades, China has made tremendous contributions to balancing capture and aquaculture rates worldwide.

### Analysis of nitrogen balance in aquaculture ecosystems in China

**Figure 3:**
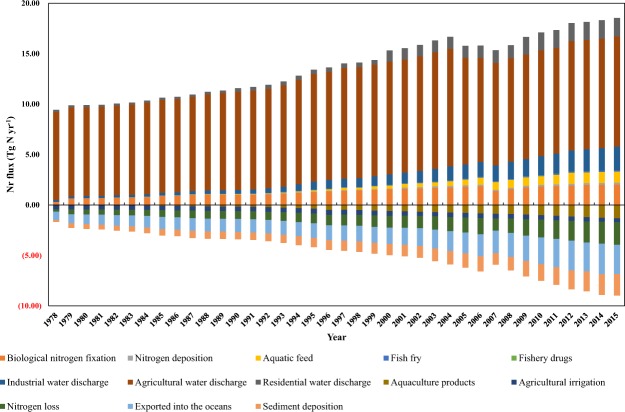
Temporal variations in the Nr balance of China’s aquaculture system for the period 1978–2015. The upper section represents the input items to the aquaculture system, and the lower section is the output items.

The increase in per capita fish consumption in China has accelerated the material recycling of aquaculture ecosystems (including inland and marine waters). Therefore, we assessed the Nr balance in the aquaculture ecosystem to determine where Nr is transported and how much Nr accumulates in the ecosystem so that efforts to reduce the environmental impact of Nr can be successfully targeted and implemented (Fig. 3). Figure 3 shows that the current disturbance of aquaculture to the entire water ecosystem in China is relatively small compared to its effects on agricultural, industrial, and domestic wastewater. The anthropogenic Nr inputs (including aquatic feed, fish fry and fishery drugs) in aquaculture ecosystems used for higher fish production increased from 0.011 Tg N yr^−1^ in 1978 to 1.22 Tg N yr^−1^ in 2015. In contrast, the discharge of wastewater from agriculture, industry, and residential activities has increased by 13.52-, 0.25- and 8.35-fold, respectively. On the one hand, wastewater discharge has accelerated the N cycle in the aquaculture ecosystem, which has accelerated the transfer of Nr flow in the food chain to a certain degree (for example, the primary producers in water ecosystems, such as algae and plankton, will grow faster). On the other hand, aquaculture is increasingly being adversely impacted by pollution from agricultural, industrial and domestic sources. Excessive Nr emissions have seriously disrupted the natural circulation of aquaculture ecosystems. In particular, the discharge of agricultural wastewater has always represented a large share of Nr wastewater discharge.

For the Nr output of aquaculture ecosystems, the output value is considerably smaller than the input value. In 1978, the output value of the ecosystem accounted for 17.87% of the input value, and in 2015, the proportion increased to 48.43%. Nr accumulation in the water body leads to eutrophication of the aquaculture ecosystems, which cannot be ignored. Compared with the average annual growth rate, the total N output of the aquaculture ecosystem has exhibited a growth rate of 4.63%, while the input has increased by 1.84%. Among the numerous outputs, the N loss (also referred to as Nr emissions), exported into oceans and sediment deposition is the primary output. Although relatively small, the output of aquaculture products has increased over 38-fold in the past few decades (1978–2015), which has made an important contribution to meeting the human demand for fish. Reasonable measures must be taken to accelerate the output value of Nr so that the gap between input and output narrows, reducing the burden and negative effect on aquaculture ecosystems.

### Environmental fate and flux of Nr in aquaculture ecosystems in China

**Figure 4:**
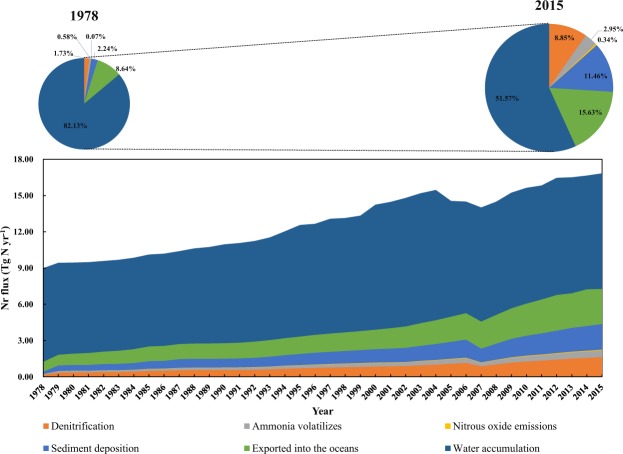
Changes in the environmental fate and flux of Nr in China’s aquaculture system for the period of 1978–2015. The area chart shows the temporal variations in each Nr environmental flux. Pie charts represent the percentage of each Nr flux in 1978 and 2015.

In recent years, the Nr accumulation in China’s aquaculture ecosystems has received considerable and unprecedented attention. Figure 1 shows that both natural and anthropogenic Nr fluxes in aquaculture ecosystems have been strengthened to varying degrees from 1978 to 2015. Figure 3 shows the source and destination of Nr streams and, more importantly, the trend of imbalance, which is the main cause of environmental problems in aquaculture ecosystems. Here, we assess the environmental fate and flux of Nr in China’s aquaculture ecosystem, as shown in Fig. 4. The figure clearly shows that most of the Nr inputs are accumulated in the aquaculture ecosystem. In 1978, water accumulation accounted for 82.13% of total Nr input. By 2015, it remained at the high level of 51.57%. Nr accumulated in water (in the form of NH_4_^+^, NH_3_, NO_2_^−^, HNO_2_, NO_3_^−^ and organic N) can stimulate or enhance the development, maintenance and proliferation of primary producers, resulting in eutrophication and seasonal algal blooms (*Microcystis* spp.) in aquaculture ecosystems. This continuous accumulation, that is, the continuous acidification process, has caused several adverse effects on aquatic plants and animals, with significant biotic damage, particularly with regard to invertebrates and fishes, in many atmospherically acidified lakes and streams. The anthropogenic eutrophication of freshwater, estuarine, and coastal marine ecosystems can cause ecological and toxicological effects that are either directly or indirectly related to the proliferation of primary producers^13^. Extensive kills of both invertebrates and fish species are probably the most dramatic manifestation of hypoxia (or anoxia) in eutrophic and hypereutrophic aquatic ecosystems with low water turnover rates^14^. The decline in productivity of aquaculture systems is in conflict with the growing demand for aquatic foods. The increase in demand is irreversible in the short term due to social and economic development, and to obtain more aquatic products, the input of anthropogenic Nr will be further increased, especially the input from aquatic feed and fishery drugs. The effect of this vicious cycle will cause further damage to aquaculture ecosystems. When Nr levels exceed the capacity of the ecosystem, the ecosystem will become a ‘dead zone’, as in the northern Gulf of Mexico^15^.

In addition, excessive input of Nr can interfere with the original N cycle of aquaculture ecosystems, which will strengthen the bacterial denitrification process to some extent^16^ (bacterial denitrification is considered the primary pathway for permanent N removal via N_2_ production^17^; N_2_ is generally considered inert and, therefore, does not react with other elements, which in turn affects the environment and human health^18^). In our study, we found that the Nr emission process of ecosystems (including denitrification, NH_3_ volatilization and N_2_O emissions) has increased nearly 10-fold in the past four decades. Even more noteworthy is that while the denitrification process can convert Nr into harmless N_2_, this process also produces by-products, N_2_O (N_2_O is a greenhouse gas, and its warming potential is 298 times that of CO_2_ (IPCC, 2007)). The production of N_2_O increased from 0.0063 Tg N yr^−1^ in 1978 to 0.063 Tg N yr^−1^ in 2015. If the N_2_O warming potential is converted to CO_2_, the equivalent CO_2_ emissions in 2015 would total approximately 29.69 million tons. In the course of the flow of water, Nr will flow with the water body and then discharge into the ocean, thus destroying the marine ecosystem. The volume of this discharge into oceans increased from 0.81 Tg N yr^−1^ in 1978 to 2.90 Tg N yr^−1^ in 2015. In addition to these processes, part of the Nr in the water, mainly organic N, is deposited in riverbeds. The growth rate of this sediment deposition process has increased by 9-fold. The Nr deposited in bottom sludge is released under appropriate conditions, resulting in the occurrence of secondary eutrophication of the ecosystem^19^.

### Consequences of alternative future scenarios for aquaculture

**Figure 5:**
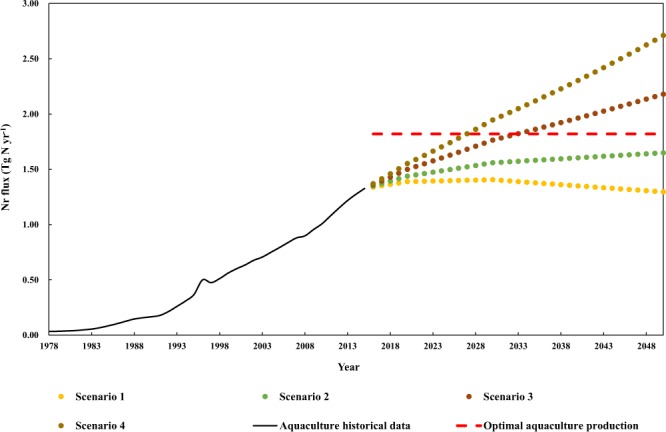
Analysis of the development trend in aquaculture in China under different scenarios. Scenarios 1–4 refer to maintaining current consumption levels, healthy and balanced diet, meeting the dietary standards of developed countries and high aquatic product consumption pattern, respectively.

To examine the impact of different human consumption levels of aquatic products on the development of aquaculture in China, we consider four scenarios and assess their prospects for aquaculture (Fig. 5). Our simulations indicate that the demand for aquaculture production will increase from −2.09% to 104.30% in 2050 over 2015 levels due to different consumption levels of aquatic products. If the current consumption level of aquatic products is maintained (Scenario 1), the demand for aquaculture production will remain relatively stable. The production will reach its peak in 2030 and decline slightly in 2050, showing a trend that aligns with population projections. For Scenarios 2–4, fish demand shows an increasing trend. Before 2030, the growth rate is faster because the population of China still shows a growing trend, and after 2030, as the population begins to decline, the growth rate slows. Considering the limitation of the ecosystem’s productivity and management effectiveness, China’s aquaculture cannot be infinitely expanded. Therefore, it is expected that the development of the aquaculture industry should reach an optimized threshold. The red dashed line in Fig. 5 shows the expected optimal value of China’s aquaculture production. Scenario 3 and Scenario 4 will exceed this optimized threshold in 2033 and 2027, respectively, and demand will probably continue to rise, causing these scenarios to further exceed these thresholds. When demand exceeds the critical threshold, the expansion of aquaculture will increase the burden on the ecosystem. At the same time, the cascade effect of Nr pollution that accompanies aquaculture will also bring negative consequences to the ecosystem and threaten human health. The destruction of ecosystems caused by environmental pollution will affect the structure, functions, and dynamics of ecosystems at all levels, which in turn will lead to the degradation of ecosystems and the loss of biodiversity, manifested as a decline in the carrying capacity and productivity of ecosystems. The contradiction between the decline in productivity and the growing demand for aquatic food will further intensify. To achieve sustainable development and coordinate the population, resources, environment and development involved in a series of major issues faced by China, appropriate changes to production, lifestyle and consumption are particularly important. The results of our simulations show that maintaining current dietary habits (Scenario 1) and implementing healthy and balanced fish consumption patterns (Scenario 2) is a promising approach.

### Towards environmentally sustainable aquaculture

Aquaculture, like other major food-producing sectors of agriculture and animal husbandry, should operate within ecological limits to minimize environmental degradation, as the environment provides ‘ecosystem services’ vital to human welfare and society’s ultimate survival as well as to that of farmed fish^20^. First, as small-scale farming households cannot afford to take high risks, the intensive management of aquaculture is the first step in the comprehensive management, improvement of production efficiency and promotion of advanced production technology. Second, improved aquaculture practises for sustainable intensification such as modern polyculture systems, recirculating aquaculture systems, integrated rice/fish farming, cage-in-pond and raceway-in-pond systems with zero effluent discharge, and integrated multi-trophic aquaculture are likely to become increasingly important^5^. Promising advanced production technologies, such as strengthening biological N fixation (BNF) and denitrification processes^21^, modern biological breeding technology, feed balanced nutritional technology, water quality treatment and regulation technology, and disease prevention and control technology^22^, are also effective ways to contribute to the sustainability of modern aquaculture. Third, the main nutrient elements (such as N and phosphorus) can be used as carriers to evaluate the three-element balance of aquatic food production, aquaculture development, and ecological and environmental protection from a life cycle perspective. Fourth, apart from aquaculture activities, other human social-economic activities, such as diet habits (the effects of changes in diet habits are shown in Fig. 5), also result in changes in nutrient cycling (especially Nr cycling). Therefore, a reasonable and balanced dietary structure is encouraged. Finally, aquaculture itself may be adversely impacted by sources of pollution, such as agricultural, industrial and domestic effluents, from the external environment (Fig. 3). Therefore, it is necessary that a national monitoring programme globally manage and monitor trends in point source pollution and diffuse agricultural pollution sources. Taking N pollution as an example, problems such as N fertilizer surplus in agriculture, industrial Nr emissions (such as those from the chemical, meat, textiles, and food processing industries), and Nr wastewater discharge from residents require attention and corresponding actions^23^.

## Discussion

Research on aquaculture at the global, national and regional scales related to economics, food security, climate change and environment aspects has been thoroughly undertaken in recent years^1,2,5,24,25^, which is conducive to understanding the spatial and temporal changes in aquaculture. Compared with these traditional aquaculture research, research using N nutrients to analyse the past, present and future trends can provide a novel and more intuitive perspective for understanding the nutritional balance and nutrient discharge in aquaculture ecosystems. This is because N is an essential element for all living organisms (N is required for the biosynthesis of key cellular components, such as proteins and nucleic acids^26^) and the main source of pollutants in aquaculture ecosystems. It is a limiting factor for primary production and biological pumps and an important medium of environmental pollution (such as eutrophication and seasonal incidents of algal blooms)^16^. The N cycle in aquaculture plays a key role in understanding how to increase the productivity of ecosystems while reducing negative environmental impacts. Aquaculture is mainly a process of the input and output of nutrient elements, and increasing the utilization efficiency of nutrients in these systems is the core scientific problem. Therefore, the importance of N balance is self-evident. In addition, N metabolism provides an effective way to quantitatively assess the impact of aquaculture systems on climate change (such as N_2_O is an important greenhouse gas) and on the extent of environmental and ecosystem damage^27^.

In research on N cycling and N footprints, the study of the N cycle in aquaculture is an important supplement. The previous large-scale studies of the N cycle and N footprint have mainly focused on specific aspects of crop farming^28,29^, livestock breeding^30^, forestry^31^ and industrial systems^32^, and in-depth studies of the historical trends in several major N flows (e.g., Haber-Bosch N fixation, BNF, lightning N fixation, and fossil fuel combustion) have been performed at global^33^, national^9,23^ and regional scales^34^. However, limited attention has been devoted to holistic and detailed studies of the N cycle in aquaculture ecosystems. Therefore, providing a high-quality N cycle analysis can provide an interesting contribution to the theory and practise of sustainable N management. As a typical case, the N footprint in China has posed important and growing impacts on human welfare and ecological protection. Addressing the issue of N pollution in the aquaculture ecosystem of China has an important role in global environmental management. Moreover, China is an important and interesting reference for other emerging countries and the international community as a whole.

Several potential improvements could be made to our methodology. First, the transfer of Nr flow in the food chain in aquaculture was not calculated because the marine food chain is extremely complex and lacks support from actual data and real-world cases. The future development of marine biology theory can provide a theoretical basis for the transfer of Nr flux in the food chain. Second, to predict the consequences of human activity (such as agriculture, industry and urban activities) in aquaculture ecosystem, there is a pressing need to understand the basic mechanisms that underlie microbial N transformations^26^. The biochemical N cycle pathways that are dominated or catalysed by microorganisms are waiting to be discovered. Third, with the advancement of globalization, trade in aquatic products will become increasingly normalized, which will impose higher requirements on aquaculture in China. Subsequent analysis of alternative future scenarios for aquaculture supply may consider the impact of trade modules. Fourth, the primary data source and the parameters in our model are based on the Statistical Yearbook and the existing literature, respectively. The Statistical Yearbook data lack a certain degree of empirical verification, and certain parameters are also considered to be the overall mean values for the entire country. Taken together, these uncertainties mean that it was difficult to accurately quantify the N flow in the model. This suggests that the measurement data in representative aquaculture ecosystems, which are roughly estimated in this study, need to be monitored during a full production cycle, as to fill in the gaps in the database^11,35^. Finally, impacts of pollution are not linearly connected to the quantity of Nr losses but are also determined by the distribution of Nr losses and vulnerable receptors in time, space and chemical form^36^. For instance, the degree of environmental impact from nitrous oxide emissions and ammonia volatilization is not the same, and a more effective assessment is worth conducting. In addition, critical environmental thresholds for aquaculture ecosystems should be defined and used as indicators to assess the degree of pollution in ecosystems to facilitate industrial adjustment and policy formulation.

## Methods

### System definition and model description

The aquaculture ecosystem in China includes inland (rivers, lakes and fish ponds) and marine waters (specific coastal waters). A national Nr cycling model was developed in China’s aquaculture ecosystem by using the substance flow analysis method, and the schematic model is shown in Fig. 1. The natural and anthropogenic processes involved in the N cycle in aquaculture ecosystems are presented in the figure. In our study, the effects of terrestrial ecosystems and human activities on aquaculture ecosystems are also considered, including wastewater discharges from agriculture, industry, and residential activities. These wastewater discharges will affect the environment and productivity of the aquaculture ecosystem. However, part of the N in the wastewater will be converted and utilized by microorganisms (six distinct nitrogen-transforming processes: assimilation, ammonification, nitrification, denitrification, anaerobic ammonium oxidation (anammox) and N fixation), thus providing primary producers with a source of bioavailable forms of N^26^. The results are aggregated to the national level to analyse Nr fate and flux across subsystems throughout the entire country.

### Flow description and data acquisition

Nr inputs for aquaculture ecosystems include BNF, N deposition, aquatic feed, fishery drugs, fish fry and the wastewater discharges from agriculture, industry, and residential activities, and the concrete function for calculating these Nr fluxes is shown in Equation (1). BNF in the aquatic ecosystem mainly involves naturally submerged plants and BNF by cyanobacteria. In an undisturbed ecosystem, BNF is performed by submerged plants and algae and provides products for fish and shrimp at higher trophic levels throughout the food chain. The rate of BNF in aquatic systems is affected by factors such as the dissolved oxygen level and photosynthetic activity^37^. The rate of BNF used in this study is from the existing literature^23,38^ (Table S1), and the area of aquaculture ecosystem in China is from the China Fisheries Yearbook^39^ (Table S8). N deposition refers to the amount of atmospheric deposition of Nr compounds (including gaseous NO_x_, HNO_3_ and NH_3_ as well as granular nitrate and ammonium salts) in the form of subsidence back to the surface. Nutrient and acid sources involved in aquatic ecosystem functions can be divided into dry deposition and wet deposition sources. In the literature^40,41^, the wet and dry deposition rates in China are listed (Table S1). The data on aquatic feed, fishery drugs and the fish fry are from the China Feed Industry Annual^42^, the China Agriculture Yearbook^43^ and the China Fisheries Yearbook^39^, respectively. N contents were obtained from scientific papers and reports^23,44^ (Table S2). The amount of wastewater discharged by agriculture, industry, and residential activities refers to the portion without treatment, which is calculated by subtracting the treatment from the amount produced. The detailed estimations and data sources of wastewater discharges from agriculture, industry, and residential activities are described in our previous research^23,45^ (Tables S3–S6), and the time span in our study is 1978 to 2015.

Nr outputs for aquaculture ecosystems include aquatic product outputs, agricultural irrigation water outputs, air emissions, sediment deposition and export into the oceans, and the specific calculation is shown in Equation (2). We estimated the Nr fluxes of aquatic products considering their total production and N concentration. Data on marine and inland water aquatic products (including fish, shrimp, crabs, shellfish, algae and other aquatic products) for the period of 1978–2015 were taken from the China Statistical Yearbook^46^, the China Fisheries Yearbook^39^ and the China Agriculture Yearbook^43^. The data on the production of aquatic products in various countries of the world are from the Fisheries and Aquaculture Statistics published by the Food and Agriculture Organization of the United Nations^12^. N contents of a variety of aquatic products were obtained from the existing literature^9,23^ (Table S2). Agricultural irrigation water output was calculated using the consumption of agricultural irrigation water and the average N content of irrigation water. The data of agricultural irrigation water consumption are from the China Statistical Yearbook^46^. The N content of water quality refers to the ‘Annual Statistical Report of the Environment’ issued by the Ministry of Environmental Protection of China^47^ (Table S7). For the air emission process in aquaculture ecosystems, the main forms are denitrification, ammonia volatilization and nitrous oxide emissions. We consider nitrous oxide emissions separately because it is an important greenhouse gas. The ratio of the various forms of emissions were reported in previous studies^9,23^, and the area of aquaculture ecosystems in China is from the China Fisheries Yearbook^39^ (Table S8). The sediment deposition of Nr means that some of the organic matter in the water, aged plankton, faeces and waste from the fishery, and uneaten bait and other nitrogen-containing compounds are stored in the sediments of the ecosystem through sedimentation or particulate matter adsorption. Sediment deposition is calculated by multiplying the estimated deposition rate by the area of the ecosystem. The rates of sediment deposition are from the existing literature^48^, and the area of the aquaculture ecosystem in China is from the China Fisheries Yearbook^39^ (Table S8). The data on flux of Nr exported into the oceans refer to the ‘Chinese Marine Environment Bulletin’ issued by the China Oceanic Information Network^49^. The uncertainty analysis in details of our study is shown as supplementary information.

1 $$\sum _{i=1}^{6}I{n}_{i}=\sum _{a=1}^{2}BN{F}_{a}+\sum _{b=1}^{2}N\,depositio{n}_{b}+{\rm{Aquatic}}\,{\rm{feed}}+{\rm{Fishery}}\,{\rm{drugs}}+{\rm{Fish}}\,{\rm{fry}}+\sum _{c=1}^{3}W{{\rm{astewater}}}_{c}$$

2 $$\sum _{j=1}^{5}Ou{t}_{j}=\sum _{d=1}^{2}{\rm{Aquatic}}\,{{\rm{product}}}_{d}+{\rm{Agricultural}}\,{\rm{irrigation}}+{\rm{Sediment}}\,{\rm{deposition}}+{\rm{Export}}\,{\rm{into}}\,{\rm{the}}\,{\rm{oceans}}+\sum _{e=1}^{3}{\rm{Air}}\,{{\rm{emissions}}}_{e}$$

Where *In*_*i*_ and *Out*_*j*_ represent the different N flow inputs and outputs, respectively. The right side of the equation represents different Nr flow terms. The key parameters for each item are shown in the supplementary information.

### Scenarios for predicting aquaculture production

Based on the schematic model, we can calculate the current per capita consumption of aquatic products in China (including aquaculture and capture, calculated as N content). The increase in population and the improvement in living standards will further increase the demand for aquatic products. The development of fisheries will be constrained by natural conditions, resources (water, land, energy, ecosystems’ productivity) and technological levels (materials, management efficiency). Especially for capture, the effects of climate change and global population growth are even greater. Merino reports that climate change is expected to decrease capture fishery production in China by approximately 3% by 2050^1^. Here, we predict the trend in changes in aquaculture production under different demand conditions. We have set up four scenarios for the per capita consumption of aquatic products. The future projection of the population is based on the National Population Development Plan issued by the China State Council (CSC)^50^.

#### Scenario 1: Maintain current consumption levels

In this scenario, we assumed that the population in China with food security would maintain its current dietary habits; this means that the per capita consumption of aquatic products will remain unchanged. Multiplying per capita consumption by the population prediction by CSC yields the total consumption of aquatic products. After deducting the amount of capture (according to Merino’s report^1^, we assume that the annual reduction in capture production remains the same, while by 2050, capture production will have fallen by 3% from 2015 levels in China), the required production for aquaculture can be obtained.

#### Scenario 2: Healthy and balanced diet

With the development of the economy and the improvement in people’s living standards, the intake of aquatic products has also increased. Rockström *et al*. reported that healthy and balanced diet should have a calorie intake of 3,000 kcal day^−1^ per capita, of which 20% comes from animal products (600 kcal day^−1^ per capita)^51^. Godfray *et al*. reported that aquatic products (including fish, shrimp, crabs, shellfish, algae and other aquatic products) have a critical role in the food system, providing people with at least 15% of their animal protein intake^52^. Based on these two reports, we assume that in 2050, the energy intake from aquatic products in China’s food consumption will be 90 kcal day^−1^ per capita. Then we calculated the total demand of aquatic products in 2050 as 2.12 Tg N yr^−1^ based on the method of N requirement per unit of caloric supply suggested by Liu *et al*.^35^. This level of per capita aquatic product consumption in 2050 is probably 120% of that in 2015. The difference between the total demand for aquatic products and the amount captured represents the demand for aquaculture, with a value of 1.65 Tg N yr^−1^.

#### Scenario 3: Meet the dietary standards of developed countries

We assume that China will reach the level of aquatic product consumption in developed countries by 2050^2,12^. The Fisheries and Aquaculture Statistics published by the Food and Agriculture Organization of the United Nations disclosed that the total amount of aquatic products for human consumption in the developed countries in 2015 reached 125,073 kilotons (approximately 3.39 Tg N yr^−1^, the N content of various aquatic products is shown in Table S2)^12^. It can be further calculated that the per capita consumption of aquatic products in developed countries is about 2.89 Tg N yr^−1^ per capita. This means that the per capita consumption in 2050 will increase by about 50% compared with 2015. With similar calculation methods, we can get future changes in aquaculture production.

#### Scenario 4: High aquatic product consumption pattern

Aquatic products can provide a wealth of protein, vitamins and trace elements, and fish have much less fat than meat and can play an extremely important role in improving the nutritional status of individuals, in particular those at risk such as children and women^3^. If the proportion of aquatic products of consumed food is increased to replace part of meat consumption, the demand for aquatic products will further increase. In countries with high consumption patterns of aquatic products (such as South Korea and Norway), the animal protein intake from aquatic products accounts for about 20–25%^53^. Here we take an average of 22.5% to calculate the future demand for China’s aquatic products. In this case, the energy intake provided by aquatic products is 135 kcal day^−1^ per capita, and the total demand for converted aquatic products is 3.18 Tg N yr^−1 ^^35,51^, which is probably about 180% of the per capita consumption of aquatic products in 2015. Therefore, we can calculate the production required for aquaculture by subtracting the amount of capture from the total demand for aquatic products.

In addition, as China’s aquaculture is faced with limited land and water resources, the aquaculture area in China, without considering the large-scale development of deep-sea breeding space or the promotion of large-scale industrial aquaculture, has reached a threshold^24^. To avoid destroying the ecological environment, we assume that China will maintain or even reduce the current aquaculture area in the next few decades. The average production per unit area of aquaculture in China is approximately 8000 kg/hm^2,25,54,55^. With the promotion of aquaculture technology, we believe that the level of aquatic product production across China can reach this level. Therefore, we estimate that the optimal value of Chinese aquaculture production, in terms of N, is 1.82 Tg N yr^−1^.

## Electronic supplementary material

Supplementary information
