# Supplementary material for: The trends of aquacultural nitrogen budget and its environmental implications in China
Source: Sci Rep. 2018 Jul 18;8:10877. doi: 10.1038/s41598-018-29214-y (PMC6052169; doi:10.1038/s41598-018-29214-y)
Supplement: Supplementary file 1 — Supplementary information [file 41598_2018_29214_MOESM1_ESM.docx]

Supplementary Information (SI)

# The trends of aquacultural nitrogen budget and its environmental implications in China

# Zhibo Luo, Shanying Hu*, Dingjiang Chen

Center for Industrial Ecology, Department of Chemical Engineering, Tsinghua University, Beijing 100084, China.

* [Corresponding author: hxr-dce@tsinghua.edu.cn](mailto:Corresponding%20author:%20hxr-dce@tsinghua.edu.cn)

The main purpose of the supplementary information is to provide the data sources, explain and document the values and rationale (such as parameters and data) used in calculations of Nr cycling China’s aquaculture ecosystem. We also describe the uncertainty associated with the calculations.

# Table Description

Table S1 shows the biological nitrogen fixation (BNF) rate and nitrogen atmospheric deposition rate in various provinces of China during the period of 1978-2015.

Table S2 shows the nitrogen content of aquatic feed, fishery drugs and the fish fry.

Table S3 and table S4 shows the nitrogen loss process, nitrogen loss rate and reference for agriculture (including cropland, grassland, forest and livestock modules).

Table S5 shows the production coefficient and emission coefficient of total N wastewater in the industrial production process.

Table S6 shows the urban and rural population in China, the proportion of fecal wastewater discharge and the wastewater discharge in daily necessities.

Table S7 shows the proportion of various types of water quality in the river basin in China and various types of water quality nitrogen content.

Table S8 shows the inland (rivers, lakes and fishponds) and marine waters (specific coastal waters) aquaculture area in China and the estimation of nitrogen loss from aquaculture ecosystem.

Table S9 shows the uncertainty snalysis of national Nr cycling in China’s aquaculture ecosystem.

# Uncertainty Analysis

In our national N cycling model of China’s aquaculture ecosystem, the main uncertainties are associated with (i) production and consumption data for N-related products (such as aquatic products, fishery drug, aquatic feed, etc.) and (ii) uncertainty in the estimated model parameter values due to a limited number of case studies. Statistical Yearbook data are generally enterprise-based data of a certain quantity; therefore, the data is associated with a certain degree of uncertainty. As a developing country, there are still some aquaculture production mode for smallholder economies in China, such as small lakes and reservoirs (areas usually ranging from 10 to 40 ha), as well as creeks and paddy rice fields^1^. This part of the relevant data may be lacking, resulting in data deviation. We wonder that the data will be relatively smaller than the actual situation. However, in recent years the consensus among studies is that the Chinese official data are the best available data^2^. A uniform method of system partitioning and more standardized data collection make the uncertainty of statistical data at a lower level. The parameter values used in this study are mainly the mean values from case studies of China, but some have been modified or replaced by similar factors from other regions because of a lack of information. In addition, some parameters are from historical data. With technological progress and innovation, these parameters will change, but we could not obtain real-time data for annual calculations (e.g. data on the N loss of leaching in agricultural production), which increased uncertainties in the estimates for Nr fluxes in N cycle model.

Based on the above analysis, we perform an uncertainty analysis with a coefficient of variation (CV) [high (±30%), median (±20%), or low (±10%)] according to these assumptions, uncertainties, and ranges of parameters (data shown in table S9)^3^. For example, the Nr flux of fishery drugs has relatively low CV because the associated N contents are broadly consistent across China; therefore, the estimated data are accurate at the national level. However, the Nr fluxes of wastewater discharge from agriculture, industry and residential activities have a relatively high CVs because they are influenced by several sectors and factors, thus affecting large-scale estimations and simulations^4^.

Table S1 The BNF rate and nitrogen atmospheric deposition rate in China between 1978 and 2015.

| Region | The BNF rates / g N m^-2^ yr^-1^ | The atmospheric deposition rate /kg N hm^-1^ yr^-1^ | | | | | | | References |
| --- | --- | --- | --- | --- | --- | --- | --- | --- | --- |
|  |  | 2015 | 2014 | 2013 | 2009-2012 | 2001-2008 | 1991-2000 | 1978-1990 |  |
| Beijing | 25 | 39.5 | 38.2 | 36.9 | 35.7 | 25.0 | 10.6 | 5 | ^5-8^ |
| Tianjin | 25 | 29.8 | 28.9 | 27.9 | 27.0 | 21.0 |  |  |  |
| Hebei | 25 | 29.8 | 28.9 | 27.9 | 27.0 | 21.0 |  |  |  |
| Shanxi | 25 | 27.6 | 26.7 | 25.9 | 25.0 | 21.0 |  |  |  |
| Inner Mongolia | 6 | 13.3 | 12.8 | 12.4 | 12.0 | 10.0 |  |  |  |
| Liaoning | 25 | 32.9 | 31.9 | 30.8 | 29.8 | 15.9 |  |  |  |
| Jilin | 25 | 16.6 | 16.0 | 15.5 | 15.0 | 14.9 |  |  |  |
| Heilongjiang | 25 | 16.6 | 16.0 | 15.5 | 15.0 | 13.2 |  |  |  |
| Shanghai | 25 | 21.9 | 21.2 | 20.5 | 19.8 | 18.2 |  |  |  |
| Jiangsu | 25 | 11.4 | 11.0 | 10.7 | 10.3 | 13.6 |  |  |  |
| Zhejiang | 25 | 7.3 | 7.1 | 6.8 | 6.6 | 18.2 |  |  |  |
| Anhui | 25 | 11.9 | 11.5 | 11.2 | 10.8 | 11.8 |  |  |  |
| Fujian | 25 | 20.0 | 19.4 | 18.7 | 18.1 | 12.8 |  |  |  |
| Jiangxi | 25 | 24.9 | 24.1 | 23.3 | 22.5 | 30.0 |  |  |  |
| Shandong | 25 | 34.2 | 33.0 | 32.0 | 30.9 | 23.6 |  |  |  |
| Henan | 25 | 27.6 | 26.7 | 25.9 | 25.0 | 4.4 |  |  |  |
| Hubei | 25 | 38.7 | 37.4 | 36.2 | 35.0 | 33.0 |  |  |  |
| Hunan | 25 | 31.3 | 30.3 | 29.3 | 28.3 | 33.0 |  |  |  |
| Guangdong | 25 | 20.0 | 19.4 | 18.7 | 18.1 | 42.0 |  |  |  |
| Guangxi | 25 | 13.3 | 12.8 | 12.4 | 12.0 | 10.1 |  |  |  |
| Hainan | 25 | 19.9 | 19.2 | 18.6 | 18.0 | 10.1 |  |  |  |
| Chongqing | 25 | 33.1 | 32.0 | 30.9 | 29.9 | 1.0 |  |  |  |
| Sichuan | 25 | 33.1 | 32.0 | 30.9 | 29.9 | 1.0 |  |  |  |
| Guizhou | 6 | 13.5 | 13.0 | 12.6 | 12.2 | 1.0 |  |  |  |
| Yunnan | 6 | 5.5 | 5.3 | 5.2 | 5.0 | 3.5 |  |  |  |
| Tibet | 6 | 1.5 | 1.5 | 1.4 | 1.4 | 1.0 |  |  |  |
| Shaanxi | 25 | 11.1 | 10.7 | 10.3 | 10.0 | 13.2 |  |  |  |
| Gansu | 6 | 9.2 | 8.9 | 8.6 | 8.3 | 8.3 |  |  |  |
| Qinghai | 6 | 5.5 | 5.3 | 5.2 | 5.0 | 5.0 |  |  |  |
| Ningxia | 6 | 9.2 | 8.9 | 8.6 | 8.3 | 8.3 |  |  |  |
| Xinjiang | 6 | 5.5 | 5.3 | 5.2 | 5.0 | 5.0 |  |  |  |

Table S2. Nitrogen content of aquatic feed, aquatic products and fishery drugs.

|  | Feed ingredients | Nitrogen content (%) | References |
| --- | --- | --- | --- |
| Aquatic feed | Corn | 1.5 | ^9-11^ |
|  | Wheat and wheat bran powder and other by-products | 2.8 |  |
|  | Rice bran | 2.0 |  |
|  | Soybean meal | 8.0 |  |
|  | Fish meal | 11.6 |  |
|  | Cottonseed meal | 7.1 |  |
|  | Rapeseed meal | 6.5 |  |
|  | Meat and bone meal | 12.0 |  |
| Aquatic products of marine waters | Fish | 3.2 | China Food Composition Table 2015^12^ |
|  | Shrimp and crab | 3.0 |  |
|  | Shellfish | 2.2 |  |
|  | Algae | 3.7 |  |
|  | Other types | 2.9 |  |
| Aquatic products of inland waters | Fish | 2.7 |  |
|  | Shrimp and crab | 2.7 |  |
|  | Shellfish | 1.8 |  |
|  | Other types | 1.2 |  |
| Fishery drugs | | 5.0 | ^13^ |

Table S3 The nitrogen loss process, nitrogen loss rate and reference for agriculture (including cropland, grassland and forest modules). Unit are in kg N ha^-1^ yr^-1^.

| Loss process  Types | Horizontal runoff | Leakage | Artificial drainage | References |
| --- | --- | --- | --- | --- |
| Dryland | 4.4 | 14.0 | - | ^14-19^ |
| Paddy field | 8.8 | 11.0 | 5.5 | ^16-18,20-22^ |
| Grassland | 10.0 | - | - | ^23-25^ |
| Evergreen broad - leaved forest | 4.4 | - | - | ^26-28^ |
| Deciduous broad - leaved forest | 3.0 | - | - |  |
| Coniferous and broad - leaved mixed forest | 2.9 | - | - |  |
| Coniferous forest | 1.3 | - | - |  |
| Shrub | 1.9 | - | - |  |
| Bamboo forest | 2.4 | - | - |  |

Table S4. Livestock feeding period, daily excrement/urine, nitrogen content (based on fresh) and unutilized proportion.

| Livestock species | Feeding cycle (d) | Excretion of fecal matter and its nitrogen content | | Urine excretion and nitrogen content | | Unutilized proportion (%) | References |
| --- | --- | --- | --- | --- | --- | --- | --- |
|  |  | Excretion (kg·d^-1^) | Nitrogen content (%) | Excretion (kg·d^-1^) | Nitrogen content (%) |  |  |
| Cow | 133.5 | 18.0 | 0.4 | 9.0 | 0.5 | 16.7 | ^29-31^ |
| Horse | 76.6 | 10.0 | 0.4 | 5.0 | 0.7 | 15.2 |  |
| Donkey | 100.0 | 10.0 | 0.5 | 5.0 | 0.2 | 22.2 |  |
| Mule | 63.7 | 10.0 | 0.3 | 5.0 | 0.2 | 22.2 |  |
| Sheep | 243.1 | 1.5 | 1.0 | 0.5 | 0.6 | 9.7 |  |
| Pig | 198.5 | 2.0 | 0.5 | 3.0 | 0.2 | 23.9 |  |
| Rabbit | 179.6 | 0.08 | 0.9 | 0.0 | - | 22.2 |  |
| Birds | 210.0 | 0.1 | 0.8 | 0.0 | - | 17.3 |  |

Table S5. The production coefficient and emission coefficient of total N wastewater in the industrial production process.

| Industry | Product | Units of coefficient | Production coefficient | Emission coefficient | References |
| --- | --- | --- | --- | --- | --- |
| Mining and smelting industry | Phosphate powder | g / ton product | 20.0 | 16.3 | Total nitrogen and phosphorus emission coefficient in key industry^2,32^ |
|  | Graphite | g / ton product | 764.0 | 424.0 |  |
| Electronic information industry | Device chip | g / per piece product | 2.9 | 0.9 |  |
|  | Integrated circuit chip | g / per piece product | 6.8 | 0.2 |  |
| Livestock and poultry slaughter, meat processing industry | Fresh pork | g / per head | 88.5 | 40.6 |  |
|  | Fresh beef | g / per head | 65.8 | 33.2 |  |
|  | Frozen lamb | g / per head | 43.0 | 26.7 |  |
|  | Frozen poultry meat | g / per head | 225.0 | 83.3 |  |
|  | Fish frozen products | g / per ton product | 3946.9 | 1694.3 |  |
|  | Meat products | g / per ton product | 1930.0 | 1020.0 |  |
| Food manufacturing industry | White wine | g / per ton product | 1332.8 | 684.1 |  |
|  | Alcohol | g / per ton product | 7862.5 | 3025.5 |  |
|  | Beer | g / one thousand liters product | 800.0 | 440.0 |  |
| Chemical industry | Synthetic ammonia | kg / per ton product | 0.9 | 0.6 |  |
|  | Coke | g / per ton product | 300.8 | 154.4 |  |
|  | Cowhide | kg / per ton product | 12.3 | 6.3 |  |
|  | Pigskin | kg / per ton product | 13.7 | 7.1 |  |
|  | Sheepskin | kg / per ton product | 13.9 | 7.4 |  |
| Textile processing industry | Cotton | g / per ton product | 1295.7 | 1170.2 |  |
|  | Yarn, thread | g / per ton product | 235.5 | 235.5 |  |
|  | Dyed cotton fabric | g / per ton product | 3277.0 | 2950.3 |  |
|  | Printed silk woven fabric | g / per ton product | 4796.9 | 4424.6 |  |
|  | Cotton textile products | g / per ton product | 2917.5 | 2641.6 |  |
|  | Hair products | g / per ton product | 980.9 | 790.6 |  |
|  | Hemp products | g / per ton product | 35.4 | 33.8 |  |
|  | Silk products | g / per ton product | 5967.6 | 4471.4 |  |
|  | Impregnated textiles | g / per ton product | 234.4 | 211.0 |  |
|  | Water wash shirt, trousers | g / per ton product | 2058.2 | 1955.8 |  |
|  | Knitwear | g / per ton product | 4433.4 | 4223.0 |  |
|  | Lint pulp | g / per ton product | 2790.6 | 2544.9 |  |
|  | Chemical Fiber | g / per ton product | 738.9 | 669.0 |  |

Table S6 The urban and rural population in China, the proportion of fecal wastewater discharge and the wastewater discharge in daily necessities.

| Year | Urban population (10,000) | Rural population (10,000) | The proportion of fecal wastewater discharge in urban (%) | The proportion of fecal wastewater discharge in rural areas (%) | Wastewater discharge in daily necessities (including synthetic detergents, chemicals, Tg N yr^-1^) | References |
| --- | --- | --- | --- | --- | --- | --- |
| 1978 | 17245 | 79014 | 5 | 5 | 0.0037 | ^2,3,6,33-38^ |
| 1979 | 18495 | 79047 | 5 | 5 | 0.0041 |  |
| 1980 | 19140 | 79565 | 5 | 5 | 0.0040 |  |
| 1981 | 20171 | 79901 | 5 | 5 | 0.0043 |  |
| 1982 | 21480 | 80174 | 5 | 5 | 0.0050 |  |
| 1983 | 22274 | 80734 | 5 | 5 | 0.0058 |  |
| 1984 | 24017 | 80340 | 5 | 5 | 0.0067 |  |
| 1985 | 25094 | 80757 | 5 | 5 | 0.0129 |  |
| 1986 | 26366 | 81141 | 5 | 5 | 0.0134 |  |
| 1987 | 27674 | 81626 | 5 | 5 | 0.0150 |  |
| 1988 | 28661 | 82365 | 5 | 5 | 0.0170 |  |
| 1989 | 29540 | 83164 | 5 | 5 | 0.0177 |  |
| 1990 | 30195 | 84138 | 14.8 | 4.9 | 0.0186 |  |
| 1991 | 31203 | 84620 | 14.8 | 4.9 | 0.0194 |  |
| 1992 | 32175 | 84996 | 14.8 | 4.9 | 0.0224 |  |
| 1993 | 33173 | 85344 | 14.8 | 4.9 | 0.0270 |  |
| 1994 | 34169 | 85681 | 14.8 | 4.9 | 0.0274 |  |
| 1995 | 35174 | 85947 | 14.8 | 4.9 | 0.0393 |  |
| 1996 | 37304 | 85085 | 14.8 | 4.9 | 0.0346 |  |
| 1997 | 39449 | 84177 | 14.8 | 4.9 | 0.0361 |  |
| 1998 | 41608 | 83153 | 14.8 | 4.9 | 0.0348 |  |
| 1999 | 43748 | 82038 | 14.8 | 4.9 | 0.0361 |  |
| 2000 | 45906 | 80837 | 24.6 | 18.7 | 0.0412 |  |
| 2001 | 48064 | 79563 | 24.6 | 18.7 | 0.0546 |  |
| 2002 | 50212 | 78241 | 24.6 | 18.7 | 0.0544 |  |
| 2003 | 52376 | 76851 | 24.6 | 18.7 | 0.0690 |  |
| 2004 | 54283 | 75705 | 24.6 | 18.7 | 0.0748 |  |
| 2005 | 56212 | 74544 | 24.6 | 18.7 | 0.0892 |  |
| 2006 | 58288 | 73160 | 24.6 | 18.7 | 0.1147 |  |
| 2007 | 60633 | 71496 | 24.6 | 18.7 | 0.1317 |  |
| 2008 | 62403 | 70399 | 24.6 | 18.7 | 0.1376 |  |
| 2009 | 64512 | 68938 | 24 | 35.7 | 0.1356 |  |
| 2010 | 66978 | 67113 | 24 | 35.7 | 0.1507 |  |
| 2011 | 69079 | 65656 | 24 | 35.7 | 0.1688 |  |
| 2012 | 71182 | 64222 | 24 | 35.7 | 0.1928 |  |
| 2013 | 73111 | 62961 | 24 | 35.7 | 0.1864 |  |
| 2014 | 74916 | 61866 | 24 | 35.7 | 0.2122 |  |
| 2015 | 77116 | 60346 | 24 | 35.7 | 0.2330 |  |

Table S7 The proportion of various types of water quality in the river basin in China and various types of water quality nitrogen content.

| Year | Class I（%） | Class II（%） | Class III（%） | Class IV（%） | Class V（%） | Lower than Class V（%） | References |
| --- | --- | --- | --- | --- | --- | --- | --- |
| N content (mg N/L Irrigation water) | 0.20 | 0.50 | 1.00 | 1.50 | 2.00 | 2.50 | The ‘Annual Statistical Report of the Environment’ issued by the Ministry of Environmental Protection of China^39^ |
| 1978-1994 | 34.44 | | 24.78 | 26.32 | | 14.47 |  |
| 1995 | 32.80 | | 23.60 | 27.70 | | 15.90 |  |
| 1996 | 32.80 | | 23.60 | 27.70 | | 15.90 |  |
| 1997 | 32.80 | | 23.60 | 27.70 | | 15.90 |  |
| 1998 | 5.40 | 24.40 | 33.00 | 13.70 | 6.60 | 16.90 |  |
| 1999 | 5.50 | 24.50 | 32.40 | 12.60 | 7.80 | 17.20 |  |
| 2000 | 4.90 | 24.00 | 29.80 | 16.10 | 8.10 | 17.10 |  |
| 2001 | 5.00 | 27.60 | 28.80 | 14.20 | 7.80 | 16.60 |  |
| 2002 | 5.60 | 33.10 | 26.00 | 12.20 | 5.60 | 17.50 |  |
| 2003 | 5.70 | 30.70 | 26.20 | 10.90 | 5.80 | 20.70 |  |
| 2004 | 6.30 | 27.20 | 25.90 | 12.80 | 6.00 | 21.80 |  |
| 2005 | 5.10 | 28.70 | 27.10 | 11.80 | 6.00 | 21.30 |  |
| 2006 | 3.50 | 27.30 | 27.50 | 13.40 | 6.50 | 21.80 |  |
| 2007 | 4.10 | 28.20 | 27.20 | 13.50 | 5.30 | 21.70 |  |
| 2008 | 3.50 | 31.80 | 25.90 | 11.40 | 6.80 | 20.60 |  |
| 2009 | 4.60 | 31.10 | 23.20 | 14.40 | 7.40 | 19.30 |  |
| 2010 | 4.80 | 30.00 | 26.60 | 13.10 | 7.80 | 17.70 |  |
| 2011 | 4.60 | 35.60 | 24.00 | 12.90 | 5.70 | 17.20 |  |
| 2012 | 5.50 | 39.70 | 21.80 | 11.80 | 5.50 | 15.70 |  |
| 2013 | 4.80 | 42.50 | 21.30 | 10.80 | 5.70 | 14.90 |  |
| 2014 | 5.90 | 43.50 | 23.40 | 10.80 | 4.70 | 11.70 |  |
| 2015 | 8.10 | 44.30 | 21.80 | 9.90 | 4.20 | 11.70 |  |

Table S8 The aquaculture area and estimation of nitrogen loss from aquaculture in China.

| Year | Inland water aquaculture area （hectares） | Marine aquaculture area （hectares） | Denitrification （Tg N yr^-1^） | NH_3_ volatile （Tg N yr^-1^） | N_2_O release （Tg N yr^-1^） | Sediment deposition (Tg N yr^-1^） | References |
| --- | --- | --- | --- | --- | --- | --- | --- |
| 1978 | 94412 | 1142959 | 0.1633 | 0.0544 | 0.0063 | 0.2116 | ^1,2,6,40-44^ |
| 1979 | 105931 | 2566551 | 0.3528 | 0.1176 | 0.0136 | 0.4570 |  |
| 1980 | 118855 | 2685041 | 0.3701 | 0.1234 | 0.0143 | 0.4795 |  |
| 1981 | 121876 | 2700147 | 0.3725 | 0.1242 | 0.0144 | 0.4826 |  |
| 1982 | 143003 | 2859879 | 0.3964 | 0.1321 | 0.0153 | 0.5135 |  |
| 1983 | 164272 | 2889878 | 0.4031 | 0.1344 | 0.0156 | 0.5223 |  |
| 1984 | 213467 | 3055703 | 0.4315 | 0.1438 | 0.0167 | 0.5590 |  |
| 1985 | 243739 | 3456876 | 0.4885 | 0.1628 | 0.0189 | 0.6328 |  |
| 1986 | 286113 | 3490813 | 0.4986 | 0.1662 | 0.0193 | 0.6459 |  |
| 1987 | 369260 | 3794940 | 0.5497 | 0.1832 | 0.0212 | 0.7121 |  |
| 1988 | 413260 | 3837093 | 0.5610 | 0.1870 | 0.0217 | 0.7268 |  |
| 1989 | 423207 | 3812333 | 0.5591 | 0.1864 | 0.0216 | 0.7243 |  |
| 1990 | 428940 | 3834727 | 0.5628 | 0.1876 | 0.0217 | 0.7291 |  |
| 1991 | 449370 | 3866010 | 0.5696 | 0.1899 | 0.0220 | 0.7379 |  |
| 1992 | 499040 | 3977140 | 0.5909 | 0.1970 | 0.0228 | 0.7654 |  |
| 1993 | 587540 | 4160430 | 0.6267 | 0.2089 | 0.0242 | 0.8119 |  |
| 1994 | 653530 | 4448980 | 0.6735 | 0.2245 | 0.0260 | 0.8725 |  |
| 1995 | 715750 | 4669340 | 0.7108 | 0.2369 | 0.0275 | 0.9209 |  |
| 1996 | 822060 | 4857960 | 0.7498 | 0.2499 | 0.0290 | 0.9713 |  |
| 1997 | 937950 | 4954837 | 0.7778 | 0.2593 | 0.0301 | 1.0077 |  |
| 1998 | 1004407 | 5080625 | 0.8032 | 0.2677 | 0.0310 | 1.0405 |  |
| 1999 | 1094946 | 5196241 | 0.8304 | 0.2768 | 0.0321 | 1.0758 |  |
| 2000 | 1243703 | 5277732 | 0.8608 | 0.2869 | 0.0333 | 1.1152 |  |
| 2001 | 1286458 | 5362302 | 0.8776 | 0.2925 | 0.0339 | 1.1369 |  |
| 2002 | 1344754 | 5469883 | 0.8995 | 0.2998 | 0.0348 | 1.1653 |  |
| 2003 | 1532152 | 5571496 | 0.9658 | 0.3219 | 0.0373 | 1.2512 |  |
| 2004 | 1617452 | 5663800 | 1.0197 | 0.3399 | 0.0394 | 1.3209 |  |
| 2005 | 1694531 | 5850488 | 1.0883 | 0.3628 | 0.0420 | 1.4098 |  |
| 2006 | 1774119 | 6018382 | 1.1577 | 0.3859 | 0.0447 | 1.4998 |  |
| 2007 | 1331478 | 4413612 | 0.8791 | 0.2930 | 0.0340 | 1.1389 |  |
| 2008 | 1578909 | 4971023 | 1.0324 | 0.3441 | 0.0399 | 1.3374 |  |
| 2009 | 1859313 | 5423825 | 1.1824 | 0.3941 | 0.0457 | 1.5317 |  |
| 2010 | 2080880 | 5564343 | 1.2784 | 0.4261 | 0.0494 | 1.6561 |  |
| 2011 | 2106382 | 5728568 | 1.3494 | 0.4498 | 0.0521 | 1.7481 |  |
| 2012 | 2180927 | 5907476 | 1.4349 | 0.4783 | 0.0554 | 1.8588 |  |
| 2013 | 2315569 | 6006130 | 1.5205 | 0.5068 | 0.0587 | 1.9698 |  |
| 2014 | 2305472 | 6080888 | 1.5783 | 0.5261 | 0.0610 | 2.0446 |  |
| 2015 | 2317763 | 6147241 | 1.6409 | 0.5470 | 0.0634 | 2.1257 |  |

Table S9 Uncertainty snalysis of national Nr cycling in China’s aquaculture ecosystem.

| Input/output | Assumed coefficient of variation (%) | Reference |
| --- | --- | --- |
| **Input pattern** | | |
| BNF | ±20 | ^5,6,8^ |
| N deposition | ±20 | ^2,3,7^ |
| Aquatic feed | ±20 | ^9-11^ |
| Fishery drugs | ±10 | ^13^ |
| Fish fry | ±20 | ^2,3,6^ |
| Agricultural wastewater discharge | ±30 | ^14-31^ |
| Industrial wastewater discharge | ±30 | ^2,32^ |
| Residential wastewater discharge | ±30 | ^2,3,6,33-38^ |
| **Output pattern** | | |
| Aquatic product | ±20 | ^2,3,6^ |
| Agricultural irrigation water | ±10 | ^39^ |
| Air emissions | ±30 | ^1,2,6,40-44^ |
| Sediment deposition | ±30 | ^41^ |
| Export into the oceans | ±20 | ^45^ |

# References

1 Zhang, Y., Bleeker, A. & Liu, J. Nutrient discharge from China’s aquaculture industry and associated environmental impacts. *Environ. Res. Lett.* **10**, 045002 (2015).

2 Gu, B. *et al.* Nitrogen footprint in China: food, energy, and nonfood goods. *Environ. Sci. Technol.* **47**, 9217-9224 (2013).

3 Cui, S., Shi, Y., Groffman, P. M., Schlesinger, W. H. & Zhu, Y. Centennial-scale analysis of the creation and fate of reactive nitrogen in China (1910–2010). *Proc. Natl. Acad. Sci. U.S.A.* **110**, 2052-2057 (2013).

4 Gu, B., Ju, X., Chang, J., Ge, Y. & Vitousek, P. M. Integrated reactive nitrogen budgets and future trends in China. *Proc. Natl. Acad. Sci. U.S.A.* **112**, 8792-8797 (2015).

5 Howard, D. L., Frea, J. I., Pfister, R. M. & Dugan, P. R. Biological nitrogen fixation in Lake Erie. *Science* **169**, 61-62 (1970).

6 Luo, Z., Hu, S., Chen, D. & Zhu, B. From Production to Consumption: A Coupled Human--Environmental Nitrogen Flow Analysis in China. *Environ. Sci. Technol.* **52**, 2025-2035 (2018).

7 Liu, X. *et al.* Enhanced nitrogen deposition over China. *Nature* **494**, 459 (2013).

8 Li, S., Jin, J. & Zhu, J. Characteristics of nutrient input/output and nutrient balance in different regions of China (in Chinese). *Scientia Agricultura Sinica* **44**, 4207-4229 (2011).

9 Delgado, C. L., Rosegrant, M. W. & Meijer, S. in *Annual meetings of the International Agricultural Trade Research Consortium (IATRC)* 18-19 (Auckland, New Zealand, 2001).

10 Delgado, C., Crosson, P. & Courbois, C. The impact of livestock and fisheries on food availability and demand in 2020. *Am. J. Agr. Econ.* **79**, 1471-1475 (1997).

11 Bellomonte, G., Costantini, A. & Giammarioli, S. Comparison of modified automatic Dumas method and the traditional Kjeldahl method for nitrogen determination in infant food. *J. Assoc. Off. Ana. Chem.* **70**, 227-229 (1986).

12 China Food Composition Table (in Chinese) ; <https://wenku.baidu.com/view/2f94e871e53a580217fcfe7f.html?re=view> (accessed April 20, 2017).

13 Luo, Z., Hu, S. & Chen, D. A historical view of nitrogen metabolism and its driving forces in China's chemical industry: Implications for cleaner production policy and practice. *J. Clean. Prod.* **187**, 308–317 (2018).

14 Halvorson, A. D., Del Grosso, S. J. & Reule, C. A. Nitrogen, tillage, and crop rotation effects on nitrous oxide emissions from irrigated cropping systems. *J. Environ. Qual.* **37**, 1337-1344 (2008).

15 Freney, J. Emission of nitrous oxide from soils used for agriculture. *Nutr. Cycl. Agroecosys.* **49**, 1-6 (1997).

16 Yang, S. *et al.* Characteristics and simulation of ammonia volatilization from paddy fields under different water and nitrogen management (in Chinese). *Transactions of the Chinese Society of Agricultural Engineering* **28**, 99-104 (2012).

17 Peng, S., Yang, S. & Xu, J. Ammonia volatilization and its influence factors of paddy field under water-saving irrigation (in Chinese). *Transactions of the Chinese Society of Agricultural Engineering* **25**, 35-39 (2009).

18 Zhang, Y., Hu, C., Dong, W., Chen, D. & Zhang, J. The influencing factors of production and emission of N2O from agricultural soil and estimation of total N2O emission (in Chinese). *Chinese Journal of Eco-Agriculture* **3**, 034 (2004).

19 Dosch, P. & Gutser, R. Reducing N losses (NH3, N2O, N2) and immobilization from slurry through optimized application techniques. *Fert. Res.* **43**, 165-171 (1995).

20 Guo, L., Ma, K. & Zhang, Y. Denitrification potential of different land-use types in Jiansanjiang District (In Chinese). *J. Agro-Environ. Sci.* **5**, 015 (2009).

21 Ma, P., Li, X. & Wang, H. Denitrification and its role in cycling and removal of nitrogen in river (In Chinese). *J. Agro-Environ. Sci.* **33**, 623-633 (2014).

22 Mou, X., Liu, X., Tong, C. & Liu, R. Effects of human disturbance on nitrification and denitrification potential in the Min River estuarine wetland (in Chinese). *China Environmental Science* **33**, 1413-1419 (2013).

23 Yan, Z. *et al.* Nitrogen cycling in grassland ecosystems in response to climate change and human activities (in Chinese). *Acta Pratacult. Sin.* **23**, 279-292 (2014).

24 Zhang, Y., Fan, Z., Yan, J., Sun, X. & Wang, Q. Effects of nitrogen addition on ammonia volatilization and nitrate leaching of a sandy grassland (in Chinese). *Chinese Journal of Ecology* **9**, 021 (2011).

25 Clark, C. M. & Tilman, D. Loss of plant species after chronic low-level nitrogen deposition to prairie grasslands. *Nature* **451**, 712-715 (2008).

26 Xi, J., Zhang, F. & You, X. Nitrogen balance of natural forest ecosystem in China (in Chinese). *Acta Ecol. Sin.* **27**, 2367-3257 (2007).

27 Steudler, P., Bowden, R., Melillo, J. & Aber, J. Influence of nitrogen fertilization on methane uptake in temperate forest soils. *Nature* **341**, 314-316 (1989).

28 Näsholm, T. *et al.* Boreal forest plants take up organic nitrogen. *Nature* **392**, 914-916 (1998).

29 Li, S. & Jin, J. Characteristics of nutrient input/output and nutrient balance in different regions of China (in Chinese). *Scientia Agricultura Sinica* **44**, 4207-4229 (2011).

30 Yang, F., Li, R., Cui, Y. & Duan, Y. Utilization and develop strategy of organic fertilizer resources in China (in Chinese). *Soil and Fertilizer Sciences in China* **4**, 77-82 (2010).

31 Guo, J. *et al.* Significant acidification in major Chinese croplands. *Science* **327**, 1008-1010 (2010).

32 Gu, B. *et al.* The role of industrial nitrogen in the global nitrogen biogeochemical cycle. *Sci. Rep.* **3**, 2579 (2013).

33 Gu, B. *et al.* The role of industrial nitrogen in the global nitrogen biogeochemical cycle. *Scientific Reports* **3**, 2579 (2013).

34 Luo, M., Ding, Z., Liao, Y. & Gan, H. Determination of Total Nitrogen Content in Woodlouse Polypeptide Tablets by Kjeldahl Method. *China Pharmacy* **25**, 029 (2013).

35 Breed, L. & Elliott, R. The Synthesis and Properties of N-Substituted Cyclotrisilazanes. *Inorganic Chemistry* **3**, 1622-1627 (1964).

36 Che, S. *et al.* A novel anionic surfactant templating route for synthesizing mesoporous silica with unique structure. *Nature Materials* **2**, 801-805 (2003).

37 Shao, Z., Wang, F., Yang, F., Zhao, F. & Tan, H. The Synthesis and Characterization of Nitric Acid Ester of Dihydroxypropyl Cellulose. *Energetic Materials* **12**, 138-142 (2004).

38 Su, P. *et al.* Determination of the nitrogen content in nitrocellulose using polarized light microscope. *Chinese Journal of Explosives & Propellants* **34**, 65-67 (2011).

39 Ministry of Environmental Protection of China. *Annual Statistical Report of the Environment (in Chinese)*, <http://www.zhb.gov.cn/gzfw_13107/hjtj/hjtjnb/> (2017).

40 Liu, Z. & Li, S. *China Fisheries Yearbook (in Chinese)*. (China Agriculture Press, 1979-2016).

41 Shu, T., Wen, Y. & Tang, Y. Cycle and Budget Balance of Nitrogen in the Cultivated Water (in Chinese). *Fisheries Science* **21**, 30-34 (2002).

42 Seitzinger, S. P. *et al.* in *The Nitrogen Cycle at Regional to Global Scales* 199-237 (Springer, 2002).

43 Fu, B. *et al.* Study on characteristics of nitrogen and phosphorus emission in typical small watershed of plateau lakes: a case study of the Fengyu River Watershed. *Acta Scientiae Circumstantiae* **35**, 2892-2899 (2015).

44 Shu, T., Wen, Y. & Tang, Y. Cycle and Budget Balance of Nitrogen in the Cultivated Water. *Fisheries Science* **21**, 30-34 (2002).

45 State Oceanic Administration, People's Republic of China. *China Marine Environment Status Report (in Chinese)*, <http://www.soa.gov.cn/zwgk/hygb/zghyhjzlgb/201712/t20171205_20159464.html> (2014).
